# In vitro plant tissue culture as the fifth generation of bioenergy

**DOI:** 10.1038/s41598-022-09066-3

**Published:** 2022-03-23

**Authors:** Omid Norouzi, Mohsen Hesami, Marco Pepe, Animesh Dutta, Andrew Maxwell P. Jones

**Affiliations:** 1 Mechanical Engineering Program, School of Engineering, University of Guelph, Guelph, ON N1G 2W1 Canada; 2 Gosling Research Institute for Plant Preservation, Department of Plant Agriculture, University of Guelph, Guelph, ON N1G 2W1 Canada

**Keywords:** Biotechnology, Bioenergy, Chemical engineering

## Abstract

Developing and applying a novel and sustainable energy crop is essential to reach an efficient and economically feasible technology for bioenergy production. In this study, plant tissue culture, also referred to as in vitro culture, is introduced as one of the most promising and environmentally friendly methods for the sustainable supply of biofuels. The current study investigates the potential of in vitro -grown industrial hemp calli obtained from leaf, root, and stem explants as a new generation of energy crop. For this purpose, the in vitro grown explants were first fully characterized in terms of elemental and chemical composition. Secondly, HTL experiments were designed by Design Expert 11 with a particular focus on biocrude. Finally, the chemical components, functional groups, and petroleum-like hydrocarbons present in the biocrude were identified by PY-GCMS. A 22.61 wt.% biocrude was produced for the sample grown through callogenesis of the leaf (CL). The obtained biocrude for CL consisted of 19.55% acids, 0.42% N compounds, 15.44% ketones, 16.03% aldehydes, 2.21% furans, 20.01% aromatics, 5.2% alcohols, and 19.88% hydrocarbons. To the best of the authors’ knowledge, this is the first report that in vitro -grown biomass is hydrothermally liquefied toward biocrude production; the current work paves the way for integrating plant tissue culture and thermochemical processes for the generation of biofuels and value-added chemicals.

## Introduction

With the world population increasing and the ongoing industrialization of our planet, there is colossal growth in the demand for clean energy^1^. Together with a finite amount of fossil fuels, this challenge encourages investigations surrounding the use of alternative energy sources that do not interfere with food security^2^. The EU's latest goal is to mandate many countries to reduce fossil fuel consumption and Greenhouse Gas (GHG) emissions to less than 2°C^3^. This transformation requires economic and technical innovations based on leading scientific practices. One solution that meets these objectives is through the replacement of fossil fuels with biofuels. So far, four generations of biofuels have been developed. For each generation, several companies have passed the demonstration plant milestone^4,5^. However, their existence in the global market heavily relies on energy subsidies and tax credit incentives^6,7^. The challenge faced by the biofuel industry is to simplify the removal of lignin from biomass, or develop a new generation of energy crop containing the lowest possible lignin content to ensure the economic feasibility of the process^8,9^. Lignin is a highly degradation-resistant phenolic polymer that hinders the thermochemical decomposition of cellulose microfibrils. In the search for low-lignin energy crops, industrial hemp (*Cannabis sativa*) has been considered an excellent feedstock for bioenergy purposes due to its desirable chemical composition^10^. Mahmud Parvez et al., 2021 compared industrial hemp's chemical composition and properties with some widely used energy crops such as spruce bark, spruce wood, willow, cereals, miscanthus, grass, and algae. Industrial hemp had lower lignin content (average 6.6 wt.%), while containing a similar carbon (48 wt.%), oxygen (43 wt.%), and hydrogen composition (6 wt.%) to selected biomass feedstocks^3^. This unique composition of industrial hemp is promising but remains insufficient to compete with fossil-fuel-based energies. Therefore, this study proposes industrial hemp in vitro culture systems to maximize and optimize applicability if this plant to the bioenergy sector. To the best of the authors’ knowledge, this is the first report using different organs and calli of in vitro grown hemp as energy sources in the Hydrothermal Liquefaction (HTL) process. In this study, the authors provide an opportunity for readers to realize the unique nature of plant tissue culture and its potential to engineer the properties of energy crops.

**Figure 1:**
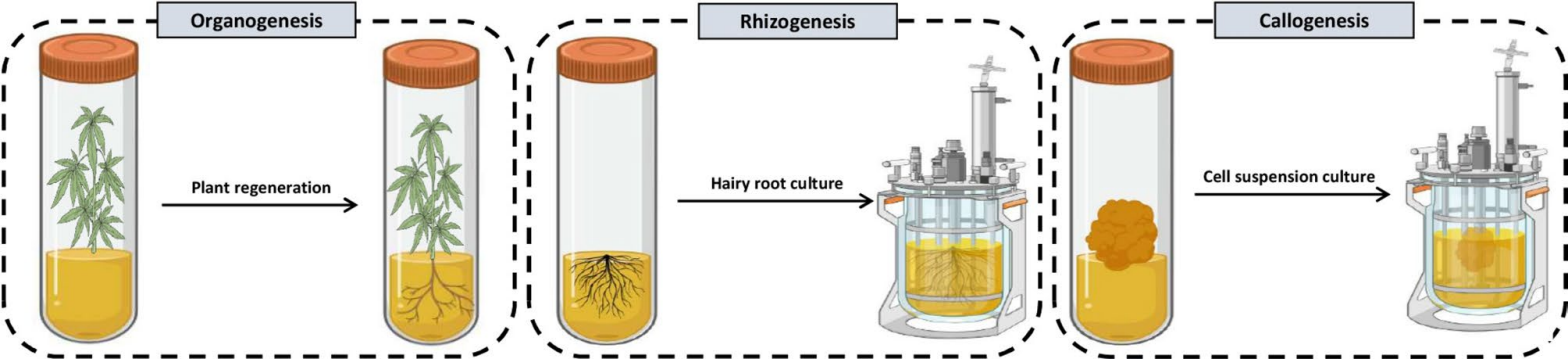
A schematic view of different in vitro culture systems.

In vitro culture is a method applied for the growth and development of plant cells, tissues, and organs that uses a nutritive culture medium under controlled sterilized conditions. This method is considered one of the most promising and environmentally friendly biotechnological practices for the sustainable supply of biofuels^11^. There are three main in vitro culture systems including organogenesis (e.g., embryogenesis, direct and indirect shoot regeneration), rhizogenesis, and callogenesis, as shown in Fig. 1. Among these methods, callogenesis can be considered a robust method for biofuel production. The callus is generally defined as an irregular bulk of parenchymatous tissue with meristematic cells that are broadly used for production of different bioactive plant molecules^11^. The main advantages of callus culture are:

- Genetic manipulation of callus for lignin engineering through transient gene transformation is much easier than other methods due to the lack of need for transgenic plant regeneration^12^.
- Somaclonal variations, which are usually seen in callus culture, can result in changes to metabolic pathways and even allows the production of new metabolites. Any phenotypic variation during callus culture is referred to as somaclonal variation that can be a result of RNA interference, histone modification, chromatin remodeling, DNA methylation, and spontaneous mutation^13^.
- Callus culture can be easily scaled up in different bioreactor systems^13^.
- Callus culture is considered a sustainable and eco-friendly process^14^.

Based on the above-aforementioned advantages, it can be concluded that callus culture has the potential to meet the exponentially growing demand for biofuel production in the near future. In this study, different parts of in vitro -grown industrial hemp seedlings were employed for callus production, and further used as a new generation of energy crop in the HTL process.

## Results and discussion

**Figure 2:**
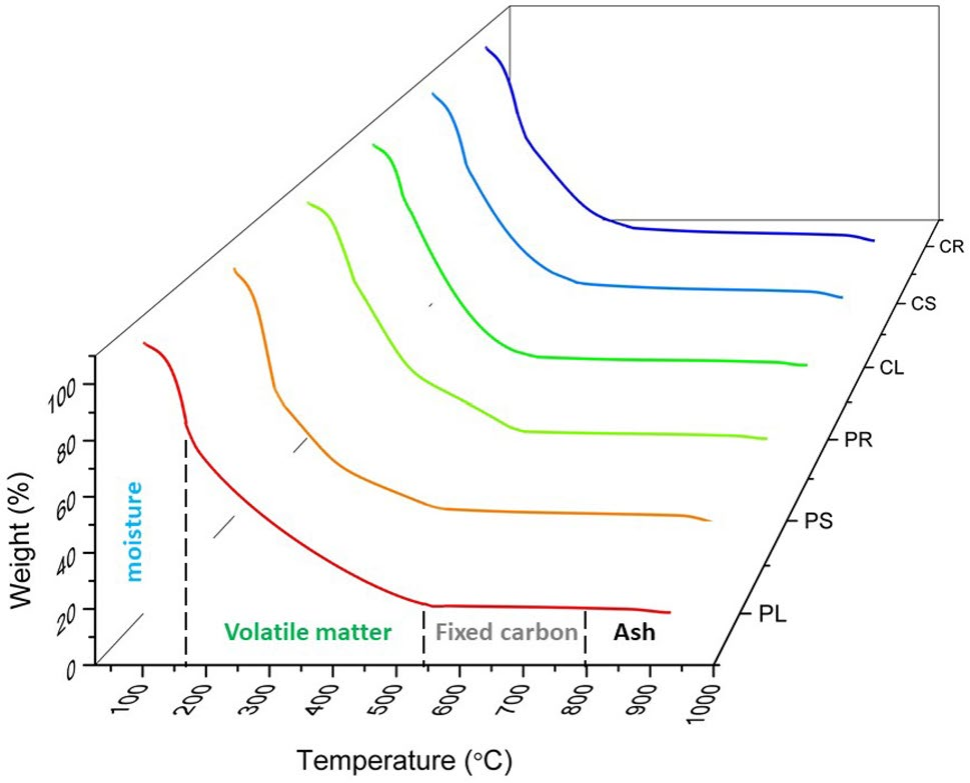
Thermogravimetric analyses of samples obtained from seed and callus culture.

Thermogravimetric analyses (TGA): The thermo-decomposition of samples was investigated by TGA, shown in Fig. 2. This technique measured the amount and rate of change in the weight of different available fractions in the samples as a function of temperature. In Fig. 2, four stages can be distinguished during the HTL of the samples: Below 160 °C, 160–540 °C, 540–800 °C, and above 800 °C. The first three regions were recorded under a nitrogen atmosphere to simulate the pyrolysis reactions. In the last region, the carrier gas was switched from nitrogen, to oxygen, to combustion reactions^20^. Loss of mass from the samples below 160 °C was due to the loss of loosely bound water, representing moisture content^21,22^. The exact values of moisture content are given in Table 1. Leaf, stem, and root obtained from in vitro -grown *Cannabis sativa* seedlings had an average moisture content of 15.45, 18.87, and 20.78 wt.%, respectively. Different calli showed lower moisture content. CL, CS, and CR's moisture contents were 10.63, 10.52, and 10.36 wt.%, respectively.

**Table 1:** Elemental and proximate analyses of samples obtained from seed and callus culture.

Samples	PL	PS	PR	CL	CS	CR
**Elemental analysis, % (w/w)**						
Carbon	50.12	49.07	42.14	55.33	51.01	52.88
Hydrogen	3.77	3.26	2.2	1.57	1.44	1.66
Nitrogen	6.41	5.51	3.2	7.35	6.52	3.61
Sulfur	0.28	0	0	0	0.33	1.65
Oxygen	22.22	28	31.56	21.52	25.19	28.68
**Proximate analysis, % (w/w)**						
Moisture	15.45	18.87	20.78	10.63	10.52	10.36
Volatile matter	50.79	45.87	39.58	61.34	58.95	58.47
Fixed carbon	16.56	21.1	23.74	15.8	15.02	15.51
Ash	17.2	14.16	15.9	12.23	15.51	15.66
HHV (MJ/Kg)	19.01	19.22	19.54	20.91	21.88	21.30

Between 160 and 800 °C, there are two regions of mass that corresponded to volatile matter and fixed carbon. The second region shows that degradable organic fractions (cellulose and hemicellulose) decompose at 160 °C. This region can be considered the active decomposition zone, in which the highest depolymerization rate takes place^23^. As shown in Fig. 2, callus-based samples had higher volatile matter values than seed-based samples. CL represents the highest recorded value, 61.34 wt.%. Beyond 800 °C, under a nitrogen atmosphere, no further mass losses were found. Therefore, at this point, the carrier gas was switched from nitrogen to oxygen to, combustion. The third and fourth regions show the fixed carbon and ash portions, respectively. In the combustion process, the combustible residue dissipated after the volatile matter distilled off, and this mass loss corresponds to the fixed carbon content. In essence, fixed carbon contains carbon, but also minor quantities of hydrogen, oxygen, and nitrogen^24^. This portion is located at the third region of TGA and is measured by subtracting the sum of moisture, volatile matter, and ash content in the sample. The weight percentage of fixed carbon in samples is in the order of PR (23.74 wt.%) > PS (21.1 wt.%) > PL (16.56 wt.%) > CL (15.8 wt.%) > CR (15.51 wt.%) > CS (15.51 wt.%), indicating that seedling-based samples had a higher fixed carbon value.

The mass left behind after 800 °C is associated with inorganic materials, or ash. These substances are not decomposed under this range of temperatures. The ash values in samples were in the order of PL (17.2 wt.%) > PR (15.9 wt.%) > CR (15.66 wt.%) > CS (15.51 wt.%) > PS (14.16 wt.%) > CL (12.23 wt.%).

For all samples, cellulose and hemicelluloses are the primary biopolymers being thermally decomposed in the temperature range of 160–540 °C. The decomposition in all samples begins with hemicellulose at 160 °C, shifting gradually to cellulose, and reaches a minimum decomposition rate at 540 °C. The differences in these six samples' inherent structures and chemical nature are due to somaclonal variations. This phenomenon usually occurs during the plant's callogenesis cycle and leads to changes in the metabolic pathway and even the production of new metabolites.

**Figure 3:**
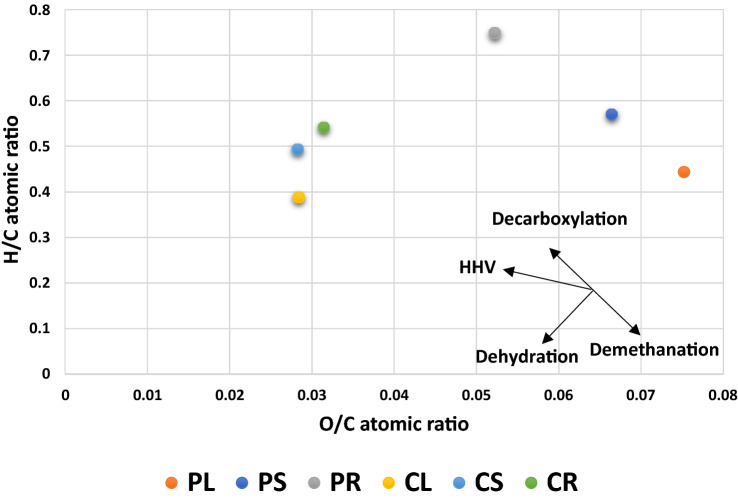
Van Krevelen diagram with samples obtained from seed and callus cultures.

Elemental analysis (CHNS-O): Results from CHNS-O analysis are presented in Table 1. As can be seen, the results agree with literature reporting the elemental analysis of naturally grown hemp^3^. In vitro production of *Cannabis sativa s*howed only minor changes in elemental composition. Compared to naturally grown hemp, in vitro produced samples had lower oxygen. For this reason, the High Heat Value (HHV) values for the samples were higher than what is typical for naturally grown hemp. Another notable observation is related to the difference in carbon content between seed and callus-based samples. The carbon content in callus-based samples was higher than seed-based content, while they contained more oxygen. Elemental analysis represented in Table 1 indicates, in all cases, that the percentage of carbon obtained from organ samples were lower than their respective callus counterpart samples. Conversely, in all cases, higher percentages of oxygen were obtained from organ samples compared to their counterpart callus samples. The H/C and O/C atomic ratios are graphed on a Van Krevelen Diagram, in Fig. 3, to investigate the potential of samples in terms of decarboxylation, demethanation, dehydration, and HHV for the HTL process. Figure 3 demonstrates that PL, PS, and PR are more inclined towards dehydration than their callus culture counterparts (CL, CS, and CR). This confirmed the TGA analysis, showing the presence of more moisture in seed-based samples than callus-based samples. The Van Krevelen Diagram also shows that PL, PS, and PR are more inclined towards decarboxylation reactions when compared to CL, CS, and CR. This can be considered a reason for higher carboxylic functional groups in bio-oil derived from seedling-based samples than callus-based samples^25^. A higher H/C ratio and lower O/C ratio indicates a higher HHV. The HHV values in samples are in the order of CS > CR > CL > PR > PS > PL, indicating the higher amount of HHV for samples produced through callus culture than in vitro grown seedlings^26,27^. To put this in context, Mahmud Parvez et al., 2021 compared industrial hemp with several biomass materials. They found hemp to have an average HHV of 18–19 MJ/kg, which was similar to the average HHV obtained for cereals and miscanthus. They found the HHV of hemp to be slightly lower than woody biomasses such as spruce wood (19–20 MJ/Kg) and spruce bark (20–23 MJ/Kg). However, the samples studied here had not only a higher HHV than the naturally grown industrial hemp, but also their HHV values were comparable to woody biomass. HHV for CS, CR, CL, PS, PR, and PL were 21.88, 21.3, 20.91, 19.54, 19.22, and 19.01, respectively.

The results presented in Table 1 raise an interesting question regarding the changes in the chemical and elemental composition of samples. These changes can be related to somaclonal variation occurring during callus culture. This phenomenon can lead to changes in metabolic pathways due to RNA interference, histone modification, chromatin remodeling, DNA methylation, and spontaneous mutation^13^. Recently, Adamek et al. (2021), through whole-genome sequencing, showed that somatic mutation in cannabis can change the metabolic pathway of cannabinoid biosynthesis^28^. We believe that 5th generation energy crops could be even more improved through somaclonal variation and/or genetic manipulation to obtain a sample with the lowest amount of lignin. Since clustered regularly interspaced short palindromic repeats (CRISPR)-mediated genome editing and *Agrobacterium*-mediated gene transformation have been developed in cannabis, genetic manipulation of callus can be considered a promising tool to change the lignin pathways in order to produce more bioenergy^29–31^. This study suggests a new approach for future research projects that utilize genetically modified crops for biofuel production.

Design of experiments: To investigate the effects of temperature on biocrude, aqueous phase, gas, and hydrochar in HTL of the samples, twenty-three experiments were designed by the I-optimal matrix of Design Expert 11. HTL experiments were conducted in a Parker autoclave engineers' reactor at three different temperatures of 250, 300, and 350 °C with a retention time of 20 min. The yields of the bioproducts obtained from HTL of the six in vitro grown samples are given in Table 2. The weight percentages of products varied from sample-to-sample. This was due to the variations in chemical composition that occurred through somaclonal variations.

**Table 2:** I-optimal design layout and experimental results.

	Factor 1	Factor 2	Response 1	Response 2	Response 3	Response 4
Run	A: Temperature	B: Samples	Gas	Biocrude	Aqueous	Hydrochar
	°C		Wt%	Wt%	Wt%	Wt%
1	250	CR	34.01	12.64	17.23	36.12
2	250	PR	22.91	13.9	13.55	49.64
3	350	PL	31.07	23.73	9.65	35.55
4	350	CL	37	22.39	12.48	28.13
5	300	CR	34.99	14.19	15.85	34.97
6	350	CR	36.77	16.41	14.65	32.17
7	300	PS	25.81	17.1	17.11	39.98
8	250	CL	36.89	19.14	11.08	32.89
9	300	CS	37.74	17	12.6	32.66
10	300	CS	38.01	17.11	12.74	32.14
11	300	CL	36.22	21.01	12.58	30.19
12	300	CR	35.1	14.25	15.76	34.89
13	250	PS	23.14	17.51	19.09	40.26
14	350	PS	30.22	20.04	13.66	36.08
15	300	PR	24.23	16.01	13.16	46.6
16	250	CS	36.07	15.5	13.53	34.9
17	350	PR	25.6	17.51	11.1	45.79
18	350	CS	39.16	17.99	12.8	30.05
19	300	CL	36.07	21.12	12.5	30.31
20	300	PL	28.31	28.2	10.5	32.99
21	250	PL	25.06	19.02	19.16	36.76
22	300	PR	25	16.78	12.34	45.88
23	300	PS	25.6	16.51	17.69	40.2

The highest biocrude yield was obtained when PL was hydroliquified at the temperature of 300 °C. The biocrude yield for PL first increased from 19.02 wt. % to 28.2 wt. %, by increasing the temperature from 250 to 300 °C, and then decreased to 23.73 wt. % at 350 °C. However, biocrude yield in CL, in the selected temperature range, increased from 21.01 to 22.39 wt. %. The lowest amount of biocrude was obtained from HTL of PR and CR, which were 12.64 and 13.9 wt. %, respectively. The hydrochar yield of the samples ranged from 28.13 to 49.64 wt.%, which varied proportionally with fixed carbon and ash content in the original samples. Both first and second maximum hydrochar yields were observed in HTL of PR at 250 and 300 °C, which were 49.64 and 46.6 wt.%, respectively. In all samples, the amount of hydrochar kept decreasing with increasing temperature from 250 to 350 °C. These trends are similar to many other reports.

In addition to finding variations in products, Design expert 11 enabled the authors to find optimum solutions for different scenarios. The constraints applied to the software are given in Table S1. In this study, the authors aimed to find the optimum operational conditions where biocrude is maximized and hydrochar is minimized. The software suggested six different solutions. As shown in Table S2, the temperature should be set at 350 °C in all six solutions. The highest desirability corresponded to the CL, where the yields of gas, biocrude, aqueous, and hydrochar were 38.4, 22.61, 10.552, and 28.48 wt. %, respectively. Desirability is in the order of CL > PL > CS > PS > CR > PR, suggesting the great potential of leaf segments in the HTL process.

**Figure 4:**
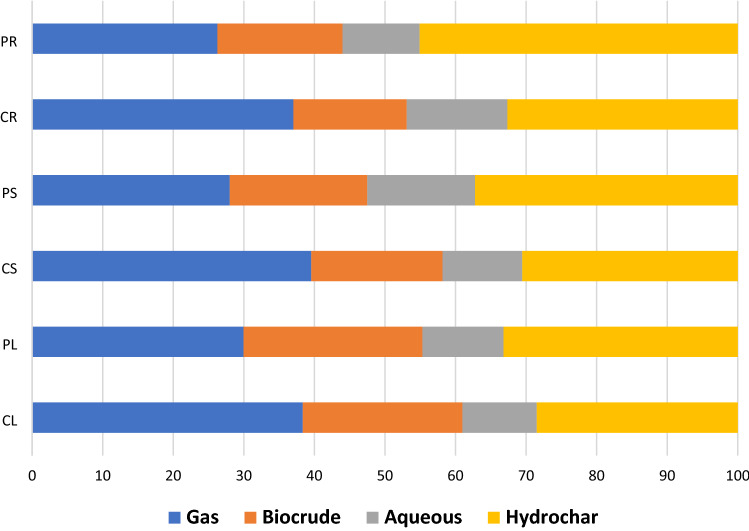
(**a**) Gas, (**b**) Biocrude, (**c**) Aqueous, and (**d**) Hydrochar yields derived from the optimum solution (350 °C and 30 min).

Figure 4 compares the percentages of bioproducts obtained from the HTL of the six in vitro samples at 350 °C. Root segments showed the lowest desirability due to their higher amount of hydrochar. As mentioned before, the higher amount of fixed carbon and ash in root segments make them prone to produce more hydrochar than other segments. In terms of hydrochar, the leaf yielded a lower amount of hydrochar than the other two segments. The char yield in both groups of samples is in the order of leaf > stem > root. Leaf segments obtained from both seedling and callus cultures showed the highest desirability. For example, 22.6 wt.% of CL were converted into the liquid portion, while the hydrochar pretty remained at its lowest limit. The gas yield in the samples is in the order of CS > CL > CR > PL > PS > PR, indicates the higher tendency of callus-based segments for gasification reactions than seedling-based samples.

**Figure 5:**
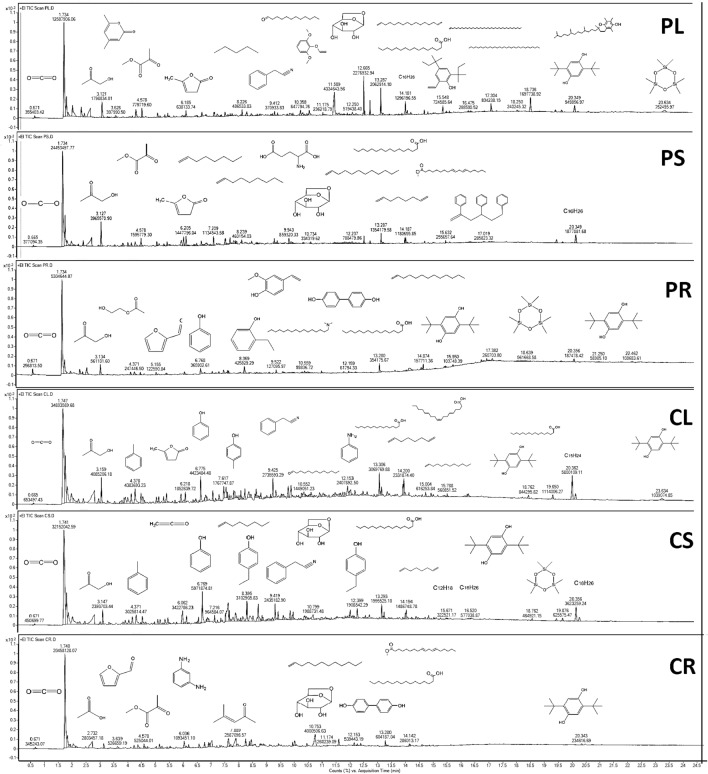
GC–MS spectra for HTL-derived bio-oils from PL, PS, PR, CL, CS, and CR.

**Figure 6:**
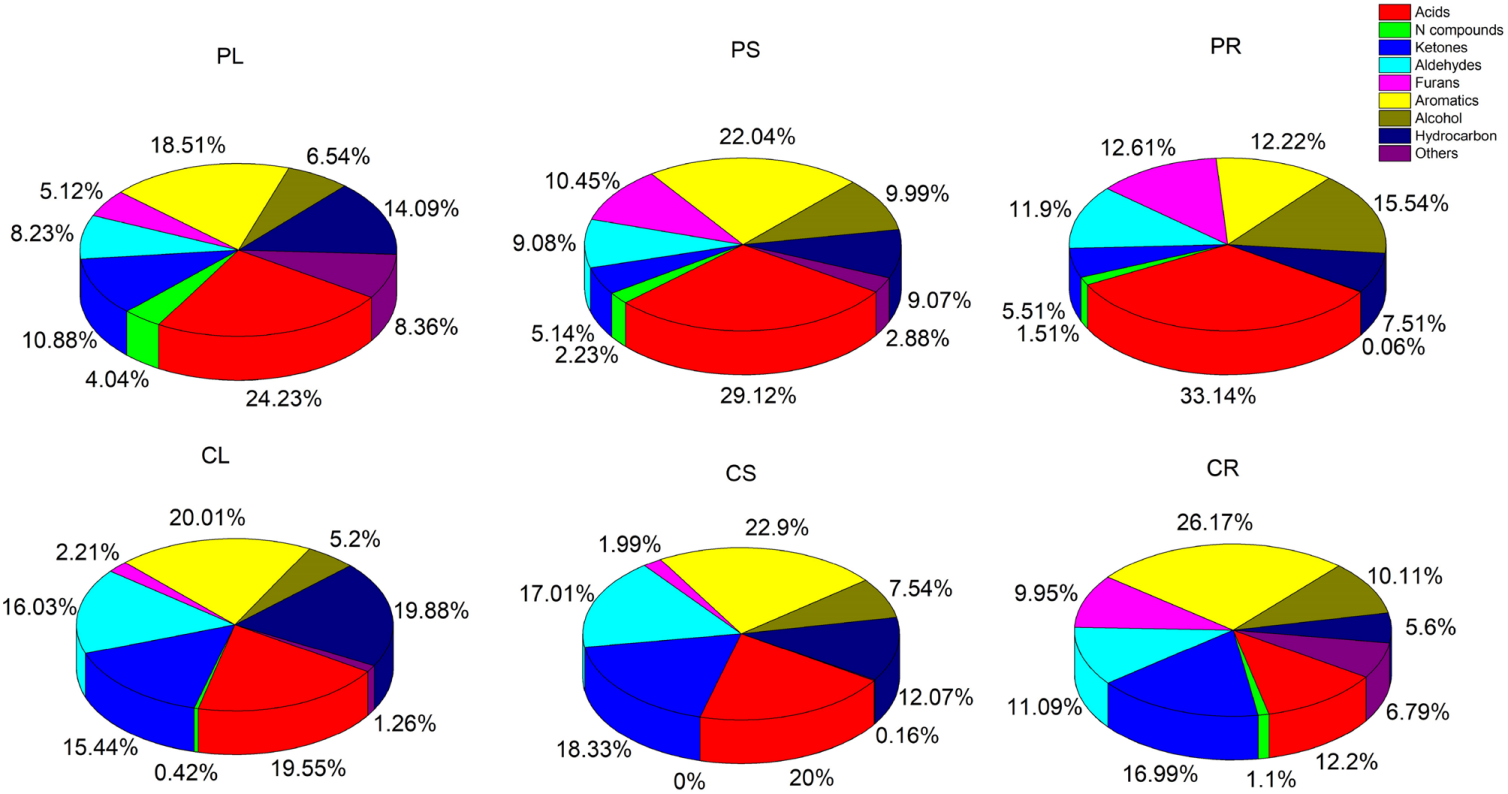
Chemical composition of hydrothermal liquefaction biocrude.

GCMS chromatograms of the biocrude: Fig. 5 compares the GCMS chromatograms of the biocrude fraction from HTL of PL, PS, PR, CL, CS, and CR. More that sixty chemicals were detected by the F-search library and are reported in Table S3. For a better interpretation, the detected chemicals were categorized into eight groups based on their functional groups: acids, hydrocarbons, aldehydes, ketones, furans, aromatics, nitrogen compounds, and alcohols^32^. The results are illustrated as a pie chart in Fig. 6.

As seen in Fig. 5, the chromatogram of the samples can be divided into four main regions based on Retention Time (RT): Below 5.5 min (I), from 5.5 to 10.5 min (II), from 10.5 to 20 min (III), and above 20 min (IV). In region I, carbon dioxide, low molecular weight acids, ketones, and furans are the main components produced by the hydrolysis of natural biopolymers and the initial fragmentation of their derivatives (C6 sugars like fructose and glucose). The most intensive peak in this region corresponds to carbon dioxide_,_ which is 19.83, 28.25, 35.07, 34.48, 18.39, and 17.91% for PL, PS, PR, CL, CS, and CR, respectively. Carbon dioxide is mostly derived from Cellulose Acetate (AC) and hydroxy butyl methyl cellulose. Unlike other lignocellulosic biomass biocrude, which produce a high selectivity of acetic acid, for PL, PS, PR, CL, and CS, this chemical did not show up. Among these six samples, acetic acid was only detected for CR (4.72%). Furans only was recorded for root segments, which are only in the form of 2-Furfural. This chemical is produced from two different sources: hydroxyethyl cellulose (HEC) and cellulose, respectively. The second region (II) is mostly rich in aromatics such as toluene, phenol, p-Creso, 2 Ethyl phenol, 4 Ethyl phenol, 2-Methoxy-4-vinylphenol, vinylsyringol, and diphenol. This type of functional group mostly comes from either dehydration of ketones or lignin composition. Levoglucosan also appears in this region, which is considered the most prevalent sugar in HTL of cellulose^33^. This compound was only produced during the HTL of CR with the high selectivity of 6.74%. The third region (III) of the chromatogram is known to possess hydrocarbons (mainly alkenes). In this region, alkenes such as 1-Dodecene, 1,15-Hexadecadiene, C16H26 (tetramer), 1,7-Octadiene, 1-Tridecene, C12H18 (trimer), C16H26 (tetramer), and 5-Hexene-1,3,5-triyltribenzene were detected. In the last region (IV), we mostly had quinones. In this study, 2,5-Di-tert-butylhydroquinone was detected for all samples. These compounds are derived from aromatic compounds by converting an even number of –CH=groups into –C(=O)– groups with any necessary rearrangement of double bonds^34^.

Chemical composition of hydrothermal liquefaction biocrude: The total peak areas of acids, N compounds, ketones, aldehydes, furans, aromatics, alcohol, and hydrocarbons are shown in Fig. 6. Since the yield of biocrude in the HTL of all samples was relatively similar, area % can be used to compare the content of the target functional group in the biocrude^35^. In all samples, acids and aromatics were the most prevalent functional groups. The selectivity of acids in samples is in the order of PR (33.14%) > PS (29.12%) > PL (24.23%) > CL (19.55%) > CS (20%) > CR (12.2%), indicating that biocrude obtained from seedling-based samples are richer in acids than callus-based samples. Another significant difference between these two groups of samples was related to the hydrocarbons. Callus-based samples showed higher selectivity for hydrocarbons. The selectivity of this functional group for CS, CR, and CL were 18.33, 16.99, and 15.44%; while PL, PS, and PR showed lower selectivity of 14.09, 9.07, and 7.51%, respectively. The balance between acids and hydrocarbons has already been reported in the literature^36^. Aliphatic acids can be converted into hydrocarbons through condensation, decomposition, and deoxygenation. Thus, further upgrading techniques are needed to decrease the corrosive nature of samples and simultaneously increase the hydrocarbon content.

Aldehydes and ketones are produced by the dehydration of C6 sugars, including glucose and fructose. The total selectivity of these two functional groups in callus-based samples was higher than seed-based samples. Among the samples, CL and CS biofuel showed superior quality. The obtained biocrude for CL consisted of 19.55% acids, 0.42% N compounds, 15.44% ketones, 16.03% aldehydes, 2.21% furans, 20.01% aromatics, 5.2% alcohols, and 19.88% hydrocarbons.

As for nitrogen-containing compounds, PL, PS, and PR showed higher selectivity for N compounds such as m-Phenylenediamine, Phenylacetonitrile, Glutamic acid, N,N-Dimethylhexadecylamine, and Aniline. NOx emissions closely originated from these compounds, thus, lower nitrogen content in the biocrude is desirable.

**Figure 7:**
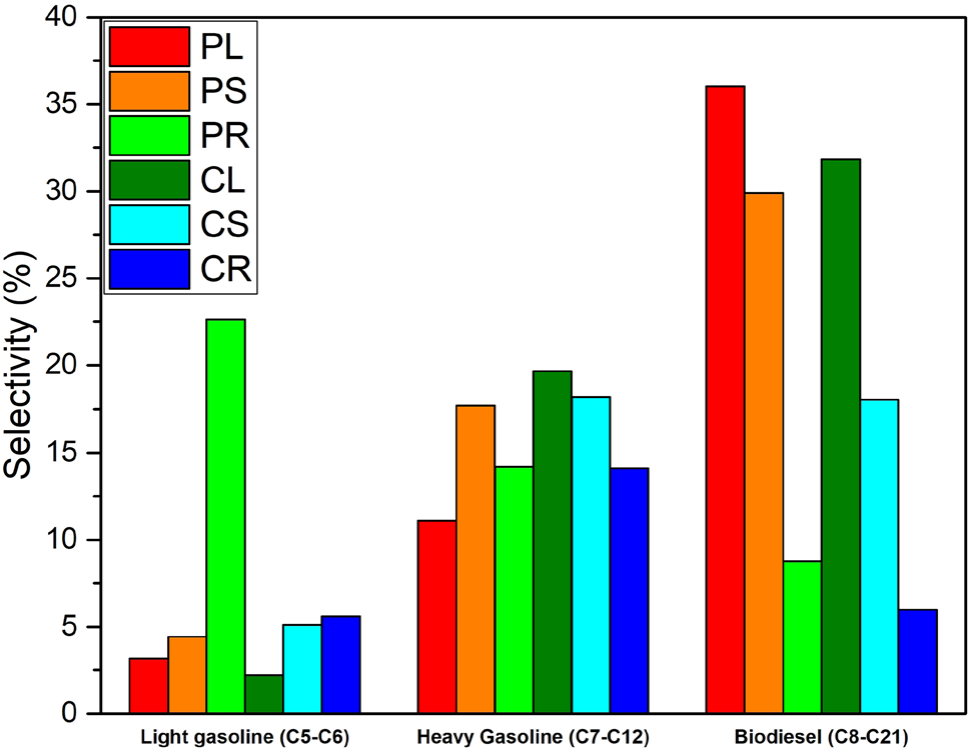
Semi-Petro based hydrocarbon fractions.

Semi-Petro based hydrocarbon fractions: The main goal of the HTL process is to upgrade biocrude towards the highest yield of semi-petro based hydrocarbon fractions. In this section, the chemicals were divided, based on their carbon number, into three groups of light gasoline, heavy gasoline, and biodiesel (Fig. 7). These three fractions of hydrocarbons relied on three consecutive reactions: Ketonization of acids, aldol condensation of ketones, and oligomerization of alkenes. Ketonization is the reaction by which two carboxylic acids can be converted into a ketone, carbon dioxide, and water^32^. Then ketones undergo the aldol condensation, followed by oligomerization, to generate fuel molecular size ranges. The selectivity of light gasoline (C5-C6) in the upgraded bio-oil is in the order of PR > CR > CS > PS > PL > CL. Although higher selectivity of C5-C6 hydrocarbon was obtained for PR, it should be noted that they are mostly in the form carboxylic acids. In the matter of C7-C12 hydrocarbons, CL showed great potential for heavy gasoline range hydrocarbons. Highest selectivity of hydrocarbons (19.88%), and lowest selectivity of alcohols (5.2%) in this feedstock makes it a great alternative for conventional feedstocks. The selectivity for heavy gasoline is in the order of CL > CS > PS > PR > CR > PL.

Most of the alkenes appeared in the third region (III) of the chromatogram and they were mostly in the range of C8-C21. That is why this fraction of hydrocarbon (biodiesel) produced by HTL Industrial Hemp explants should be given more attention. Among three different segments evaluated here, HTL of leaf and stem produced more biodiesel than HTL of root. The selectivity of biodiesel for PL, PS, PR, CL, CS, and CR were 39.03%, 29.91%, 8.77%, 31.82%, 18.04%, and 5.99%, respectively.

### Experimental section

**Figure 8:**
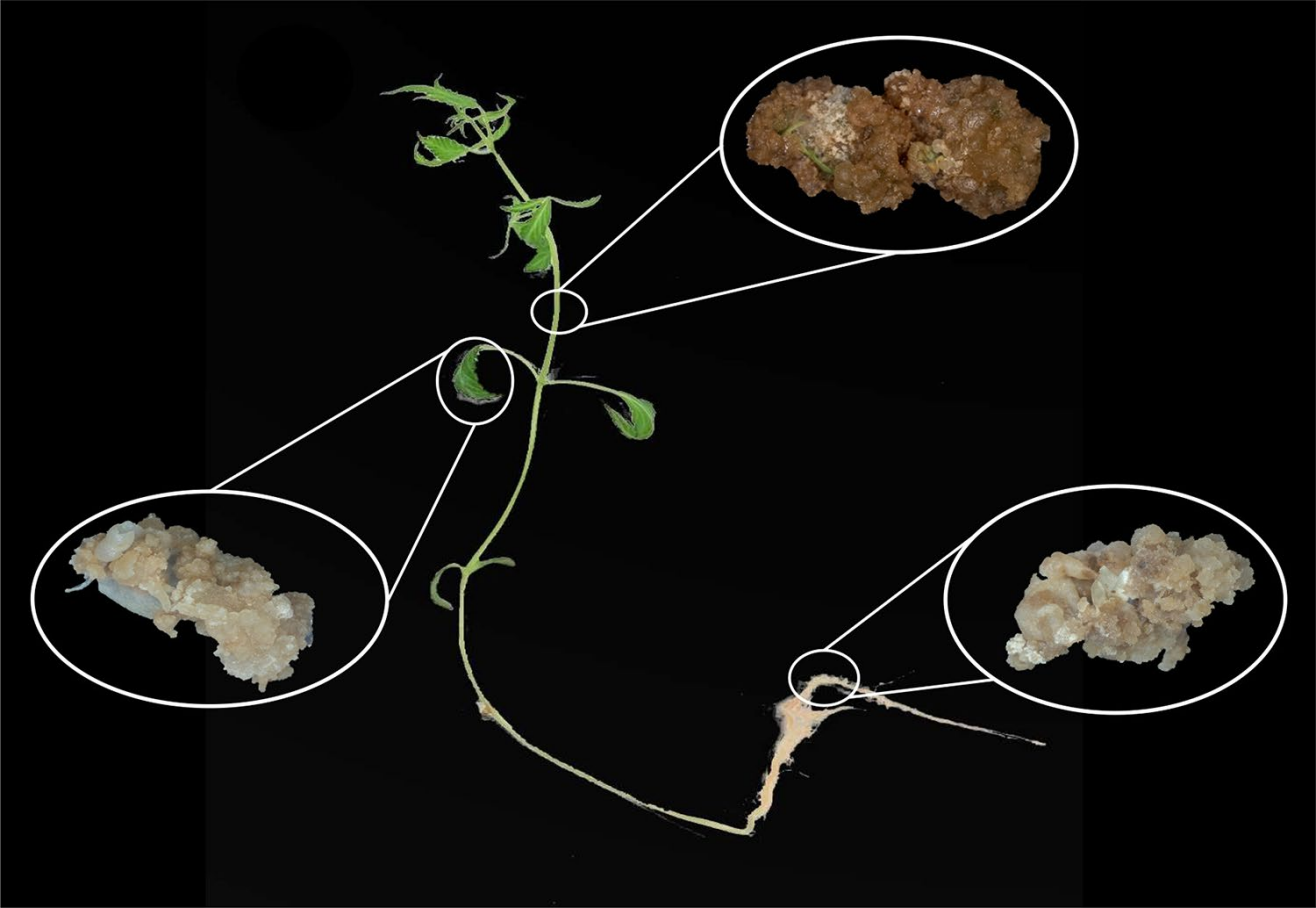
Calli obtained from different parts (leaf, stem, and root) of in vitro grown-cannabis seedling.

All the experiments done on plants are in compliance with relevant institutional, national, and international guidelines and legislation. In vitro cell culture of hemp: Industrial hemp seeds (*Cannabis sativa* cv. "Finola"; CSGA No.1 Certified seed, Lot #: 1908–18,637-17-KKF-01) were employed to obtain in vitro -grown seedlings based on the Hesami et al., (2021) protocol^15^. Briefly, after dipping the seeds in 70% ethanol for 60 s followed by 12% (v/v) commercial bleach for 12 min and three times washing with sterilized deionized water for 5 min each, the decontaminated seeds (2 seeds per box) were inoculated in 0.43 strength mMS (Murashige and Skoog Medium, Van der Salm modification (Van der Salm et al., 1994)) (Phytotech Labs, Kansas, USA) medium supplemented with 2.3% sucrose^16^. In the current study, leaf, root, and stem segments of in vitro grown plantlets of Finola cultivar were selected to investigate callogenesis based on the Hesami and Jones (2021) protocol^17^. The basal medium in the callogenesis experiment was MS (Murashige and Skoog, 1962) salt mixture medium (Phytotechnology Laboratories) with B_5_ (Gamborg et al., 1968) vitamin medium (Phytotechnology Laboratories), supplemented with 3% sucrose, 0.78 mg/l 2,4-dichlorophenoxyacetic acid (2,4-D, Fisher Scientific), and 1.32 mg/l kinetin (Fisher Scientific)^17,18^. For both in vitro seed germination and callogenesis, all media had 0.6% agar (Thermo-Fisher Scientific, Waltham, MA) and the pH of the media was adjusted to 5.8 before autoclaving for 20 min at 120 ◦C. Thirty mL of media were poured into a Magenta GA7 box (Fisher Scientific, NJ, USA). All culture boxes were placed in a growth chamber at 25 ± 2 °C under 16-h Photoperiod with 40 ± 5 μmol m^−2^ s^−1^ light intensity. Different parts of in vitro grown seedling (leaf, root, and stem) and different calli (leaf-derived callus, root-derived callus, and stem-derived callus) were used for further experiments (Fig. 8).

Hydrothermal liquefaction experiments: The hydrothermal liquefaction experiments were carried out using a high-pressure reactor (300 ml) manufactured by Parker Hastelloy. The reactor was equipped with a very precise temperature controller and magnetic stirrer (variable speed adjustment). The schematic of the reactor can be found in the authors’ previous study^19^.

Characterization of samples obtained from seed and callus culture: Proximate characteristics of samples was obtained using a TGA machine (SDT-Q600, TA instruments-Waters LLC, New Castle, USA) with a heating rate of 10 °C/min, a maximum temperature of 1000 °C, and a nitrogen flow rate of 50 mL/min. The elemental composition of the samples was obtained using a Flash 2000 elemental analyzer (Thermo Fisher Scientific, Waltham, MA, USA). The analysis was performed on a dry basis, and the oxygen content of given samples was calculated by difference: %O = 100 – (%C + %H + %N + %S + % ash).

Characterization of bio-products: The biocrude from the HTL of samples was analyzed by Frontier LAB pyrolyzer linked to an Agilent 7890B GC and the Agilent 240 Ion Trap Mass Spectrometer (Agilent Technologies, USA). 200–400 µg of samples were weighed in a U-shaped quartz tube and placed in the sample holder. Samples were flash pyrolyzed at a temperature of 300 °C, a heating rate of 20 °C/sec, with a holding period of 20 s. The Helium (He) was employed as an inert pyrolysis gas with a 20 ml/min flow rate and a split ratio of 20:1. The pyrolysis products were separated on the Agilent HP-5 MS column. The column had dimensions of 30 m × 0.25 mm with a film thickness of 0.25 μm. Chromatographic peaks were validated using the NIST library. The peaks were then sorted as per the peak area percentage with individual retention times and integrated with the help of Qualitative Analysis version 10.0 software. The compounds were further identified and sorted according to molecular formula and structure using F-search engine software.

## Conclusion

The recounted study shows the great potential of tissue culture as a suitable and sustainable method for energy crop production. The novel use of industrial hemp callus resulted in a high-quality biocrude mainly comprised of ketones and alkenes. This is mainly due to somaclonal variations that occurred throughout the callogenesis process. The yield of light gasoline, heavy gasoline, and biodiesel for PL, under optimal conditions, were 3.17%, 11.1%, and 36.03%, respectively. HTL biocrude production from industrial hemp using this plant tissue culture method offers the opportunity to integrate advanced plant agriculture and bioenergy to create a circular economy. The fifth generation of biofuel has the following advantages: First, genetic manipulation of callus enables us to engineer the lignin content. Second, the engineered calli samples produce high-quality biofuel. Finally, callus cultures have no known negative impact on ecosystems and the environment, by avoiding competition for arable land and the food and feed industry.

## Supplementary Information

Supplementary Information.

## Data Availability

All data generated or analysed during this study are included in this published article and its supplementary information file.

## Abbreviations

PL: Leaf obtained from in vitro-grown plantlets of hemp
PS: Stem obtained from in vitro-grown plantlets of hemp
PR: Root obtained from in vitro-grown plantlets of hemp
CL: Callus of leaf segments
CS: Callus of stem segments
CR: Callus of root segments
HTL: Hydrothermal liquefaction
TGA: Thermogravimetric analyses
Py-GCMS: Pyrolysis–gas chromatography-mass spectrometry
HHV: High heating value
RT: Retention time
HEC: Hydroxyethylcellulose
