# Supplementary material for: In vitro plant tissue culture as the fifth generation of bioenergy
Source: Sci Rep. 2022 Mar 23;12:5038. doi: 10.1038/s41598-022-09066-3 (PMC8943187; doi:10.1038/s41598-022-09066-3)
Supplement: Supplementary file 1 — Supplementary Information. [file 41598_2022_9066_MOESM1_ESM.docx]

**In vitro plant tissue culture as the fifth generation of bioenergy**

**Omid Norouzi^1^, Mohsen Hesami^2^, Marco Pepe^2^, Animesh Dutta^1*^, and Andrew Maxwell P. Jones^2**^**^1^Mechanical Engineering Program, School of Engineering, University of Guelph, Guelph, Ontario N1G 2W1, Canada

^2^Gosling Research Institute for Plant Preservation, Department of Plant Agriculture, University of Guelph, Guelph, Ontario N1G 2W1, Canada

[* Corresponding authors:

Bioenergy (A. Dutta), Email: [adutta@uoguelph.ca](mailto:adutta@uoguelph.ca)

Plant tissue culture (A. M. P. Jones), Email: [amjones@uoguelph.ca](mailto:amjones@uoguelph.ca)]

Table S1. Constraints

| Name | Goal | Lower Limit | Upper Limit |
| --- | --- | --- | --- |
| A: Temperature | is in range | 250 | 350 |
| Gas | none | 22.91 | 39.16 |
| Biocrude | maximize | 12.64 | 28.2 |
| Aqueous | none | 9.65 | 19.16 |
| Hydrochar | minimize | 28.13 | 49.64 |

Table S2. Solutions

| Number | Temperature | Samples | Gas | Biocrude | Aqueous | Hydrochar | Desirability |
| --- | --- | --- | --- | --- | --- | --- | --- |
| **1** | **350.000** | **CL** | **38.357** | **22.612** | **10.552** | **28.480** | **0.794** |
| 2 | 350.000 | PL | 29.958 | 25.347 | 11.495 | 33.200 | 0.790 |
| 3 | 350.000 | CS | 39.557 | 18.597 | 11.309 | 30.538 | 0.583 |
| 4 | 350.000 | PS | 28.004 | 19.487 | 15.279 | 37.230 | 0.504 |
| 5 | 350.000 | CR | 37.029 | 16.069 | 14.264 | 32.638 | 0.417 |
| 6 | 350.000 | PR | 26.247 | 17.747 | 10.929 | 45.078 | 0.264 |

Table S3. Top chemical compounds identified in the bio-oils by GC–MS along with their most-likely biopolymer source.

| N | RT | Compound | Formula | Polymer | PL | PS | PR | CL | CS | CR |
| --- | --- | --- | --- | --- | --- | --- | --- | --- | --- | --- |
| 1 | 0.665 | Carbon dioxide | CO2 | Cellulose acetate; CA | 0.56 | 0.44 | 1.7 | 0.34 | 0.26 | 0.58 |
| 2 | 1.734 | Carbon dioxide | CO2 | Hydroxy butyl methyl cellulose | 19.83 | 28.25 | 35.07 | 34.48 | 18.39 | 17.91 |
| 3 | 2.732 | Acetic acid | C2H4O2 | Cellulose acetate; CA |  |  |  |  |  | 4.72 |
| 4 | 3.121 | Hydroxy acetone | C3H6O2 | Hydroxypropyl cellulose | 2.83 | 4.58 | 3.71 | 2.1 | 1.37 |  |
| 5 | 3.626 | 4,6-Dimethyl-2H-pyran-2-one | C7H8O2 | Cellulose acetate butyrate; CAB | 0.63 |  |  |  |  |  |
| 6 | 3.639 | 2-Furfural | C5H4O2 | Cellulose |  |  |  |  |  | 0.89 |
| 7 | 4.371 | 2-Hydroxyethyl acetate | C4H8O3 | Cellulose acetate butyrate; CAB |  |  | 1.64 |  |  |  |
| 8 | 4.371 | Toluene | C7H8 | Hexabromocyclododecane |  |  |  | 2.26 | 1.73 |  |
| 9 | 4.578 | Methyl pyruvate | C4H6O3 | Cellulose | 1.23 | 1.84 |  |  |  | 0.88 |
| 10 | 5.155 | 2-Furfural | C5H4O2 | Hydroxyethyl cellulose; HEC |  |  | 0.81 |  |  |  |
| 11 | 6.036 | m-Phenylenediamine | C6H8N2 | Polypyrrole |  |  |  |  |  | 1.84 |
| 12 | 6.062 | Ketene | C2H2O | Poly (vinyl acetate) |  |  |  |  | 1.96 |  |
| 13 | 6.185 | 5-Methylfuran-2(3H)-one | C5H6O2 | Cellulose | 1.01 | 1.67 |  |  |  |  |
| 14 | 6.218 | Phenol | C6H6O | Cellulose acetate; CA |  |  | 2.42 | 2.28 | 3.41 |  |
| 15 | 7.209 | 1-Octene | C8H16 |  |  | 1.31 |  |  | 0.55 |  |
| 16 | 7.617 | p-Cresol | C7H8O | Oligomer of methylated phosphazene |  |  |  | 0.91 |  |  |
| 17 | 7.889 | Isobutenyl methyl ketone | C6H6O | 2,2,4,4-Tetramethyl-7-oxa-3,20-diazadispiro-[5.1.11.2]-heneicosan-21-one |  |  |  |  |  | 4.36 |
| 18 | 8.226 | n-Pentane | C5H12 |  | 0.77 |  |  |  |  |  |
| 19 | 8.239 | 1-Nonene | C9H18 |  |  | 0.53 |  |  |  |  |
| 20 | 8.369 | 2-Ethylphenol | C8H10O |  |  |  | 2.82 |  |  |  |
| 21 | 8.395 | 4-Ethylphenol | C8H10O | Polysulfone |  |  |  |  | 1.77 |  |
| 22 | 9.412 | Phenylacetonitrile | C8H7N |  | 0.58 |  |  | 1.41 | 1.39 |  |
| 23 | 9.522 | 2-Methoxy-4-vinylphenol | C9H10O2 | Lignin |  |  | 0.84 |  |  |  |
| 24 | 9.943 | Glutamic acid | C5H9NO4 |  |  | 0.99 |  |  |  |  |
| 25 | 10.358 | Dodecanal | C12H24O |  | 1.02 |  |  |  |  |  |
| 26 | 10.552 | Tridecanal | C13H26O |  |  |  |  | 0.75 |  |  |
| 27 | 10.559 | N,N-Dimethylhexadecylamine | C18H39N |  |  |  | 0.66 |  |  |  |
| 28 | 10.734 | Levoglucosan | C6H10O5 | Cellulose |  | 0.39 |  |  |  | 6.74 |
| 29 | 11.174 | Vinylsyringol | C10H12O3 | Lignin | 0.37 |  |  |  |  |  |
| 30 | 11.174 | 1-Dodecene | C12H24 |  |  |  |  |  |  | 0.44 |
| 31 | 11.589 | Levoglucosan | C6H10O5 | Cellulose | 6.83 |  |  |  |  |  |
| 32 | 12.153 | Aniline | C6H7N |  |  |  |  | 1.24 |  |  |
| 33 | 12.153 | Diphenol | C12H10O2 |  |  | 0.41 |  |  |  | 0.91 |
| 34 | 12.237 | 1-Dodecene | C12H24 |  |  | 0.91 |  |  |  |  |
| 35 | 12.25 | Levoglucosan | C6H10O5 | Novon | 0.82 |  |  |  |  |  |
| 36 | 12.399 | 4-Ethylphenol | C8H10O |  |  |  |  |  | 1.09 |  |
| 37 | 12.665 | 1,15-Hexadecadiene | C16H30 | Glycerides, | 3.59 |  |  |  |  |  |
| 38 | 13.28 | Palmitic acid | C16H32O2 |  | 3.25 | 1.56 | 2.34 | 1.58 | 1.14 | 1.02 |
| 39 | 14.142 | Methyl 9,12-octadecadienoate | C19H34O2 | Methyl O-acetylricinolate |  |  |  |  |  | 0.48 |
| 40 | 14.181 | C16H26 (tetramer) | C16H26 |  | 2.04 |  |  |  |  |  |
| 41 | 14.187 | 1,7-Octadiene | C8H14 |  |  | 1.36 |  | 1.2 | 0.85 |  |
| 42 | 14.874 | 1-Tridecene | C13H26 |  |  |  | 1.04 |  |  |  |
| 43 | 15.004 | Oleic acid | C18H34O2 |  |  |  |  | 0.32 |  |  |
| 44 | 15.548 | 4,6-Bis(tert-pentyl)-2-vinylphenol | C18H28O |  | 1.14 |  |  |  |  |  |
| 45 | 15.632 | Methyl 9,12-octadecadienoate | C19H34O2 |  |  | 0.3 |  |  |  |  |
| 46 | 15.671 | C12H18 (trimer) | C12H18 |  |  |  |  |  | 0.18 |  |
| 47 | 15.788 | 1-Dodecene | C12H24 |  |  |  |  | 0.29 |  |  |
| 48 | 15.95 | 2,5-Di-tert-butylhydroquinone | C14H22O2 |  |  |  | 0.69 |  |  |  |
| 49 | 16.475 | 1-Tetracontene | C40H80 |  | 0.45 |  |  |  |  |  |
| 50 | 16.52 | C16H26 (tetramer) | C16H26 |  |  |  |  |  | 0.33 |  |
| 51 | 17.019 | 5-Hexene-1,3,5-triyltribenzene | C24H24 |  |  | 0.33 |  |  |  |  |
| 52 | 17.304 | n-Hentriacontane [CH3(CH2)29CH3] | C31H64 |  | 1.31 |  |  |  |  |  |
| 53 | 17.382 | Hexamethylcyclotrisiloxane | C6H18O3Si3 |  |  |  | 1.72 |  |  |  |
| 54 | 18.25 | 2,5-Di-tert-butylhydroquinone | C14H22O2 |  | 0.38 |  |  |  |  |  |
| 55 | 18.639 | Hexamethylcyclotrisiloxane | C6H18O3Si3 |  |  |  | 3.71 |  |  |  |
| 56 | 18.736 | alpha-Tocopherol | C29H50O2 |  | 2.67 |  |  |  |  |  |
| 57 | 18.762 | 2,5-Di-tert-butylhydroquinone | C14H22O2 |  |  |  |  | 0.43 | 0.27 |  |
| 58 | 19.65 | Palmitic acid | C16H32O2 |  |  |  |  | 0.57 |  |  |
| 59 | 19.876 | Hexamethylcyclotrisiloxane | C6H18O3Si3 |  |  |  |  |  | 0.36 |  |
| 60 | 20.343 | 2,5-Di-tert-butylhydroquinone | C14H22O2 |  | 1.5 |  | 1.24 |  |  | 0.4 |
| 61 | 20.349 | C16H26 (tetramer) | C16H26 |  |  | 2.17 | 0.39 | 2.98 | 2.07 |  |
| 62 | 23.634 | Hexamethylcyclotrisiloxane | C6H18O3Si3 |  | 1.19 |  |  |  |  |  |
| 63 | 23.634 | 2,5-Di-tert-butylhydroquinone | C14H22O2 |  |  |  |  | 0.53 |  |  |
